# Putting an end to the *Pseudomonas aeruginosa* IQS controversy

**DOI:** 10.1002/mbo3.962

**Published:** 2019-10-30

**Authors:** Pierre Cornelis

**Affiliations:** ^1^ Department of Bioengineering Sciences Laboratory of Microbiology Vrije Universiteit Brussel Brussels Belgium; ^2^ Laboratoire de Microbiologie Signaux et Microenvironnement Normandie Université Université de Rouen Normandie Évreux France

**Keywords:** aeruginaldehyde, IQS, *Pseudomonas aeruginosa*, pyochelin, quorum sensing

## Abstract

Despite published evidence that IQS (2‐(2‐hydroxylphenyl)‐thiazole‐4‐carbaldehyde) is in fact aeruginaldehyde, a by‐product of the siderophore pyochelin biosynthesis or degradation and that the *ambABCDE* genes are not responsible for IQS synthesis, several authors, including in top review journals, perpetuate the wrong information. I hope that this short comment will clarify the situation once and for all.

## Graphical abstract

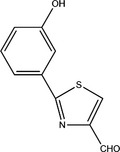

## 1 COMMENTARY

*Pseudomonas aeruginosa* is an important opportunistic pathogen causing a range of infections in immunocompromised individuals and in those with cystic fibrosis where it can establish itself in the lungs (Lyczak, Cannon, & Pier, 2000). The production of the majority of *P. aeruginosa* virulence factors is dependent on three linked quorum sensing systems, Las, Rhl, and PQS (Williams & Camara, 2009). Las and Rhl systems rely on *N*‐acyl‐homoserine lactones signal molecules whereby the Las system controls the Rhl system (Williams & Camara, 2009). A third system, involving the Pseudomonas quinolone signal (PQS) molecule, is also hierarchically dependent of Las (Williams & Camara, 2009). In Lee et al., 2013 published an article suggesting that there is a fourth quorum sensing signal molecule, termed IQS (2‐(2‐hydroxylphenyl)‐thiazole‐4‐carbaldehyde) (Lee et al., 2013), reviewed in 2015 (Lee & Zhang, 2015). They found that the production of pyocyanin, a phenazine whose production depends on the Rhl and PQS systems (Williams & Camara, 2009) is still observed in a *las* mutant under low phosphate condition (Lee et al., 2013). By screening a transposon mutant library, they found that the Las‐independent pyocyanin production was abolished in an *ambB* mutant (Lee et al., 2013). They further claimed that the *ambABCDE* genes are responsible for the biosynthesis of IQS (Lee et al., 2013). This last claim is wrong as explained in this commentary.

### 1.1 The *ambABCDE* genes are not involved in IQS synthesis

The *ambABCDE* genes code for proteins involved in the production of the antimetabolite L‐2‐amino‐4methoxy‐trans‐3‐butenoic acid (AMB) (Rojas Murcia et al., 2015) and could therefore not possibly be responsible for IQS production. AmbB and AmbE are nonribosomal peptide synthetases whose primary product is the l‐Ala‐l‐Glu‐l‐Ala tripeptide. AmbC and AmbD further modify the L‐Glu into AMB. AMB is released after removal of the two flanking L‐Ala residues and is excreted via AmbA, a LysE amino acid transporter (Rojas Murcia et al., 2015). AMB is produced by *P. aeruginosa* and causes a germination arrest in plants (Chahtane et al., 2018).

### 1.2 IQS is aeruginaldehyde, a by‐product of the siderophore pyochelin

A first indication that IQS could not be the product of AmbABCDE was the discovery that the IQS structure is identical to the structure of aeruginaldehyde, a compound produced by other Pseudomonas, including *P. protegens* and in *Burkholderia thailandensis* which do not have the *amb* genes cluster (Trottmann, Franke, Ishida, García‐Altares, & Hertweck, 2019; Ye et al., 2014). Aeruginaldehyde has been detected together with other compounds (aeruginoic acid, aeruginol, dihydroaeruginoic acid), which may be all products derived from the siderophore pyochelin biosynthetic pathway (Ye et al., 2014). In a very recent study, Trottmann et al. confirmed that aeruginaldehyde is derived from pyochelin since an incubation of the siderophore at 30°C resulted in the appearance of aeruginaldehyde (Trottmann et al., 2019). Ye et al. (2014) suggested that aeruginaldehyde could derive from dihydroaeruginoic acid (Dha) released from PchE during the assembly of the NRPS enzymes and the biosynthetic process (Ye et al., 2014). Interestingly, Maspoli, Wenner, Mislin, and Reimmann (2014) found in *P. aeruginosa* PA14 a genomic island containing genes for the biosynthesis of (enantio)pyochelin. Complementation of a pyochelin negative mutant with one NRPS gene led to the production of Dha (Maspoli et al., 2014). In a recent study, Sun et al. found that only the Rhl and PQS QS systems are involved in the regulation of phenazine production in a *P. aeruginosa* strain while mutants with deletions in *amb* or *pch* genes clusters were not affected in their production of phenazine compounds, excluding their possible participation in the QS circuitry (Sun, Zhou, Jin, Jiang, & He, 2016).

## 2 CONCLUSION

Despite the evidence presented here in this short commentary, still scientists publish articles, including reviews, mentioning IQS as the fourth quorum sensing molecule of *P. aeruginosa* being the product of AmbABCDE. The aim of this short comment is to clarify this point.

## CONFLICT OF INTEREST

None declared.

## AUTHOR CONTRIBUTIONS

PC: Conceptualization, Formal analysis, Writing—original draft, Writing—review and editing.

## ETHICAL APPROVAL

None required.

## Data Availability

Not applicable.
